# Excitability and Burst Generation of AVPV Kisspeptin Neurons Are Regulated by the Estrous Cycle Via Multiple Conductances Modulated by Estradiol Action^1^,^2^,^3^

**DOI:** 10.1523/ENEURO.0094-16.2016

**Published:** 2016-06-07

**Authors:** Luhong Wang, Richard A. DeFazio, Suzanne M. Moenter

**Affiliations:** 1 Department of Molecular and Integrative Physiology, University of Michigan, Ann Arbor, MI 48109; 2 Department of Obstetrics and Gynecology, University of Michigan, Ann Arbor, MI 48109; 3 Department of Internal Medicine, University of Michigan, Ann Arbor, MI 48109

**Keywords:** burst, GnRH, Kisspeptin, persistent sodium current, steroid, T-type calcium current

## Abstract

The preovulatory secretory surge of gonadotropin-releasing hormone (GnRH) is crucial for fertility and is regulated by a switch of estradiol feedback action from negative to positive. GnRH neurons likely receive estradiol feedback signals via ERα-expressing afferents. Kisspeptin neurons in anteroventral periventricular nucleus (AVPV) are thought to be critical for estradiol-positive feedback induction of the GnRH surge. We examined the electrophysiological properties of GFP-identified AVPV kisspeptin neurons in brain slices from mice on the afternoon of diestrus (negative feedback) and proestrus (positive feedback, time of surge). Extracellular recordings revealed increased firing frequency and action potential bursts on proestrus versus diestrus. Whole-cell recordings were used to study the intrinsic mechanisms of bursting. Upon depolarization, AVPV kisspeptin neurons exhibited tonic firing or depolarization-induced bursts (DIB). Both tonic and DIB cells exhibited bursts induced by rebound from hyperpolarization. DIB occurred similarly on both cycle stages, but rebound bursts were observed more often on proestrus. DIB and rebound bursts were both sensitive to Ni^2+^, suggesting that T-type Ca^2+^ currents (*I*_T_s) are involved. *I*_T_ current density was greater on proestrus versus diestrus. In addition to *I*_T_, persistent sodium current (*I*_NaP_) facilitated rebound bursting. On diestrus, 4-aminopyridine-sensitive potassium currents contributed to reduced rebound bursts in both tonic and DIB cells. Manipulation of specific sex steroids suggests that estradiol induces the changes that enhance AVPV kisspeptin neuron excitability on proestrus. These observations indicate cycle-driven changes in circulating estradiol increased overall action potential generation and burst firing in AVPV kisspeptin neurons on proestrus versus diestrus by regulating multiple intrinsic currents.

## Significance Statement

The brain controls fertility via the hypothalamo–pituitary–gonadal axis. Gonadotropin-releasing hormone (GnRH) neurons form the final output pathway but are not directly sensitive to critical elements of gonadal steroid feedback. Kisspeptin neurons of the anteroventral periventricular nucleus may convey steroid feedback to GnRH neurons. We studied how action potential firing of kisspeptin neurons varies between two critical phases of the estrous cycle: diestrus when estradiol exerts negative feedback to suppress GnRH release; and proestrus when estradiol feedback activates GnRH neurons. Increased spontaneous and burst firing on proestrus was observed and attributable to estrous cycle-dependent changes in multiple ionic currents. These changes were specifically driven by estradiol. This estrous cycle regulation of kisspeptin neuron excitability is likely a critical aspect of female fertility.

## Introduction

Gonadotropin-releasing hormone (GnRH) neurons form the final common pathway for central regulation of reproduction. GnRH stimulates the pituitary gland to secrete follicle-stimulating hormone and luteinizing hormone (LH) to regulate gonadal functions. Gonadal steroids provide feedback to regulate GnRH release. In males and during most of the female reproductive cycle, sex steroids suppress GnRH neuron activity and release (Karsch et al., 1987; Moenter et al., 1990; Lubbers et al., 1998; Christian et al., 2005). In females, sustained elevations in estradiol levels during the follicular phase result in a switch of estradiol action from negative to positive feedback, inducing GnRH neuron activation, and a preovulatory surge of GnRH and LH release (Christian and Moenter, 2010). Although regulated by steroid feedback, GnRH neurons do not express detectable levels of most steroid receptors, including estrogen receptor α (ERα; Hrabovszky et al., 2001), which mediates estradiol negative and positive feedback (Couse et al., 1999; Christian et al., 2008). Steroid-sensitive afferents thus likely transmit feedback signals to GnRH neurons. Estradiol-sensitive kisspeptin-expressing neurons in the arcuate nucleus and anteroventral periventricular nucleus (AVPV) may convey estradiol-negative and estradiol-positive feedback to GnRH neurons, respectively (Oakley et al., 2009).

Kisspeptin is a potent stimulator of GnRH neuron activity and release (Han et al., 2005; Pielecka-Fortuna et al., 2008; Glanowska et al., 2012). AVPV kisspeptin neurons express ERα (Smith et al., 2005), as well as GABA and glutamate (Cravo et al., 2011), both of which also excite GnRH neurons (DeFazio et al., 2002; Kuehl-Kovarik et al., 2002). AVPV kisspeptin neurons synapse on GnRH neurons (Kumar et al., 2015; Yip et al., 2015) and express elevated cFos levels during the GnRH/LH surge (Smith et al., 2006). Infusion of anti-kisspeptin antibodies into the preoptic area (Kinoshita et al., 2005), or deletion of ERα specifically from kisspeptin neurons both block estradiol-induced LH surges (Dubois et al., 2015). Together, these observations suggest a role for AVPV kisspeptin neurons in conveying estradiol-positive feedback to GnRH neurons.

Given the evidence for a role for AVPV kisspeptin neurons in estradiol-positive feedback, a fundamental question is how the firing activity of AVPV kisspeptin neurons shifts between estrous cycle stages to increase the release of kisspeptin, glutamate, and/or GABA during positive feedback. AVPV kisspeptin neurons express several ionic conductances that may shape firing patterns (Piet et al., 2013; Zhang et al., 2013b, 2015), some of which are regulated by the reproductive cycle (Piet et al., 2013), but the mechanisms that underlie changes in AVPV kisspeptin neuron excitability and the steroids underlying cycle-dependent shifts are not fully understood. In particular, the release of neuropeptides such as kisspeptin is classically linked with high-frequency bursts of action potentials (van den Pol, 2012).

We examined the spontaneous activity of AVPV kisspeptin neurons and the contributions of candidate currents to the firing properties of these cells during two estrous cycle stages: the afternoon of diestrus (di) representing estradiol negative feedback and the afternoon of proestrus (pro) representing positive feedback (Andrews et al., 1981; Gallo and Bona-Gallo, 1985). We then determined the specific circulating sex steroids that mediate the cycle-dependent changes in firing patterns and how different ionic conductances contribute to this.

## Material and Methods

### Animals

Kiss1-hrGFP mice (Cravo et al., 2011) were propagated in our colony. All mice were provided with water and Harlan 2916 chow *ad libitum*, and were held on a 14/10 h light/dark cycle with lights on at 4:00 A.M. Eastern Standard Time. Mice were used during the diestrous or proestrous phases of the estrous cycle determined by monitoring vaginal cytology of female mice 60-90 days old for at least a week before the experiments. Uterine mass was determined after brain slice preparation to confirm uteri on proestrus were >100 mg, indicating exposure to endogenous high estradiol (Shim et al., 2000). To examine the role of specific sex steroids, similarly aged adult female mice were ovariectomized (OVX) under isoflurane anesthesia (Abbott Laboratories) and were either simultaneously implanted with a SILASTIC (Dow Corning) capsule containing 0.625 µg of estradiol suspended in sesame oil (OVX+E) or not treated further (OVX). Bupivacaine was provided local to the incisions as an analgesic. These mice were studied 2-3 d after undergoing ovariectomy during the time of estradiol positive feedback (Christian et al., 2005). On the day of study, some OVX+E mice received a subcutaneous injection at 9:00 to 10:00 A.M. Eastern Standard Time of progesterone (300 μg/20 g; OVX+E+P; Dror et al., 2013), or sesame oil vehicle (OVX+E+V). All mice were killed at 3:00 to 4:00 P.M. Eastern Standard Time. There were no differences observed between OVX+E and OVX+E+V mice for firing patterns and burst generation; these data were combined for analyses, and only OVX+E mice were included for further studies to reduce animal use. All animal procedures were performed in accordance with the regulations of the University of Michigan animal care committee.

### Slice preparation and cell identification

All chemicals were purchased from Sigma-Aldrich, unless noted. All solutions were bubbled with 95% O_2_/5% CO_2_ throughout the experiments and for at least 30 min before exposure to tissue. The brain was rapidly removed and placed in ice-cold sucrose saline solution containing the following (in mm): 250 sucrose, 3.5 KCl, 26 NaHCO_3_, 10 d-glucose, 1.25 NaHPO_4_, 1.2 MgSO_4_, and 3.8 MgCl_2_, at pH 7.6 and 345 mOsm. Coronal (300 µm) slices were cut with a VT1200S Microtome (Leica Biosystems). Slices were incubated in a 1:1 mixture of sucrose saline and artificial CSF (ACSF) containing (in mm) 135 NaCl, 3.5 KCl, 26 NaHCO_3_, 10 d-glucose, 1.25 Na_2_HPO_4_, 1.2 MgSO_4_, and 2.5 CaCl_2_, at pH 7.4 and 305 mOsm, for 30 min at room temperature (∼21 to 23°C); and then were transferred to 100% ACSF for an additional 30-180 min at room temperature before recording. For recording, slices were placed into a chamber continuously perfused with ACSF at a rate of 3 ml/min with oxygenated ACSF heated to 31 ± 1°C with an inline-heating unit (Warner Instruments). GFP-positive AVPV kisspeptin neurons were identified by brief illumination at 488 nm on an Olympus BX51WI microscope. Recorded cells were mapped to an atlas (Paxinos and Franklin, 2001) to determine whether any trends based on anatomical location emerged; no such trends were apparent in these datasets. Recordings were performed from 1 to 3 h after brain slice preparation. No more than three cells per animal were included for analysis of the same parameter, and at least five animals were tested per parameter.

### Electrophysiological recording

Recording micropipettes were pulled from borosilicate capillary glass (type 7052, 1.65 mm outer diameter; 1.12 mm inner diameter; World Precision Instruments) using a Flaming/Brown P-97 puller (Sutter Instruments) to obtain pipettes with a resistance of 3-5 MΩ for whole-cell recordings, and 2-3 MΩ for targeted extracellular recordings when filled with the appropriate pipette solution. Recording pipettes were wrapped with Parafilm to reduce capacitive transients. Recordings were made with an EPC-10 dual-patch clamp amplifier and Patchmaster software (HEKA Elektronik) running on a Macintosh computer.

### Extracellular recordings

Targeted extracellular recordings were made to obtain the firing properties of cells under control conditions and with receptors for ionotropic GABA_A_, and glutamate synaptic transmission antagonized with a combination of picrotoxin (100 μm), aminophosphonovalerate (APV; 20 μm), and 6-cyano-7-nitroquinoxaline-2,3-dione (CNQX; 10 μm). This method was used because it maintains internal milieu and has a minimal impact on the firing rate of neurons (Alcami et al., 2012). Recording pipettes were filled with HEPES-buffered solution containing (in mm) 150 NaCl, 10 HEPES, 10 glucose, 2.5 CaCl2, 1.3 MgCl2, and 3.5 KCl, at pH 7.4 and 310 mOsm, and low-resistance (22 ± 3 MΩ) seals were formed between the pipette and neuron after first exposing the pipette to the slice tissue in the absence of positive pressure. Recordings were made in voltage-clamp mode with a 0 mV pipette holding potential, and signals were acquired at 20 kHz and filtered at 10 kHz. Resistance of the loose seal was checked frequently during first 3 min of recordings to ensure a stable baseline, and also before and after a 10 min recording period; data were not use if seal resistance changed >30% or was >25 MΩ. The first 5 min of this 10 min recording were consistently stable among cells, and were thus used for analysis of firing rate and burst properties.

### Whole-cell recordings

For whole-cell patch-clamp recording, two pipette solutions were used. Most recordings were performed with a physiologic pipette solution containing the following (in mm): 135 K gluconate, 10 KCl, 10 HEPES, 5 EGTA, 0.1 CaCl_2_, 4 MgATP, and 0.4 NaGTP, at pH 7.2 with NaOH and 305 mOsm. A cesium-based pipette solution, in which cesium gluconate replaced potassium gluconate, was used to reduce potassium currents and allow better isolation of calcium currents. All potentials reported were corrected on-line for liquid junction potential of −15.7 or −15.0 mV for the physiologic or Cs^+^-based solution, respectively (Barry, 1994). For all whole-cell recordings, ACSF contained picrotoxin, APV, and CNQX, as detailed above.

After achieving a minimum 1.6 GΩ seal and the whole-cell configuration, membrane potential was held at −70 mV between protocols during voltage-clamp recordings. Series resistance (R_s_), input resistance (R_in_), holding current (*I*_hold_), and membrane capacitance (C_m_) were frequently measured using a 5 mV hyperpolarizing step from −70 mV (mean of 16 repeats, 20 ms duration). Only recordings with an R_in_ of >500 MΩ, *I*_hold_ of −40 to 10 pA, R_s_ of <20 MΩ, and a stable C_m_ were used for analysis. R_s_ was further evaluated for stability, and any voltage-clamp recordings with ΔR_s_ of >15% before and after the recording protocols were excluded from analysis; current-clamp recordings with ΔR_s_ of >20% were excluded. There was no difference in *I*_hold_, C_m_, or R_s_ among any comparisons.

### Current-clamp recordings

Depolarizing and hyperpolarizing current injections (−50 to +50 pA for 500 ms, in 5 pA steps) were applied to cells from an initial voltage of −71± 2 mV, close to their basal membrane potential of −68.8 ± 1.9 mV (DeFazio et al., 2014). In a small subset of experiments (*n* = 12), the initial voltage was adjusted to −65 and −75 mV to test the voltage dependence of depolarization-induced burst-firing patterns. Tetrodotoxin (TTX; 1 μm) was used to block action potentials and reveal underlying changes in membrane potential. NiCl_2_ (100 μm), ZD7288 (50 μm), and 4-aminopyridine (4-AP; 5 mm) were applied to test the role of T-type calcium, hyperpolarization-activated mixed cation, and A-type potassium current conductance (*I*_A_) in generating bursts, respectively.

### Voltage-clamp protocols for T-type Ca^2+^ current

ACSF containing antagonists of ionotropic GABA_A_ and glutamate receptors with TTX (2 µm) and Cs-based internal solution were used for all recordings to isolate calcium currents. Two voltage protocols were used to isolate T-type Ca^2+^ currents (*I*_T_). First, total calcium current activation was examined. Inactivation was removed by hyperpolarizing the membrane potential to −110 mV for 350 ms (not shown in Fig. 4*E*). Next a 250 ms prepulse of −110 mV was given. Then membrane potential was varied in 10 mV increments for 250 ms from −110 to −30 mV. Finally, a test pulse of −40 mV for 250 ms was given. From examination of the current during the test pulse, it was evident that no sustained (high-voltage activated) calcium current was activated at potentials more hyperpolarized than −40 mV. To remove high voltage-activated (HVA) contamination from the step to −30 mV, a second protocol was used in which removal of inactivation (−110 mV, 350 ms) was followed by a 250 ms prepulse at −40 mV, then a step for 250 ms at −30 mV, and finally a test pulse of −40 mV for 250 ms. *I*_T_ was isolated by subtracting the trace following the −40 mV prepulse from those obtained after the −110 mV prepulse for the depolarized variable step to −30 mV; raw traces from the initial voltage protocol were used without subtraction for variable steps from −110 to −40 mV because of the lack of observed activation of HVA at these potentials. Activation of *I*_T_ was assessed from the resulting family of traces by peak current during the variable step phase. Inactivation of *I*_T_ was assessed from the peak current during the final −40 mV test pulse. For a subset of recordings (*n* = 3 cells), NiCl_2_ (100 μm) was used to block current generated to confirm it was *I*_T._

### Voltage-clamp ramp protocols for persistent sodium current

Physiologic pipette solution was used for voltage-clamp ramp recordings. A voltage ramp from −80 to −20 mV at 10 mV/s was used under control conditions and following TTX (2 µm) application to characterize the voltage dependence and magnitude of a TTX-sensitive persistent sodium current (I_NaP_). To test the relative roles of I_NaP_ and I_A_ in cells that did not show rebound firing, current during the ramp was quantified under control conditions, then following I_A_ block with 4-AP (5 mm) followed by the subsequent addition of TTX (2 µm).

### Data analysis

Data were analyzed off-line using custom software written in IgorPro version 6.31 (Wavemetrics) or MATLAB version 8.4 (MathWorks). For targeted extracellular recordings, the mean firing rate in hertz was determined over 5 min of stable recording. Parameters for the identification of bursts were chosen based on the distributions of interspike intervals and were confirmed by measuring the interspike interval of bursts that were identified manually using other criteria (upshift of baseline and progressive decrease of amplitude). Spikes were considered to form a burst if the interspike intervals were <105 ms. Spikes detected after an interval >105 ms were considered to be the start of a new burst or single spike. Bursts were automatically detected and confirmed by eye with false-positive detection errors manually corrected (Gaskins and Moenter, 2012).

Action potential parameters were quantified for the first action potential evoked in a firing train with minimal current injection (rheobase) from −70 mV. First spike latency was the time from the onset of current injection to the peak of the first spike. The rate of rise was the maximal slope during the rising phase of the action potential. The action potential threshold was defined as the membrane potential at which the derivative exceeded 2 V/s. Full-width at half-maximum (FWHM) was the width of the spike at the half-maximal spike amplitude from threshold. The afterhyperpolarization (AHP) amplitude was the difference between the threshold and the most hyperpolarized potential after the spike. AHP time was the delay from threshold to the peak (most hyperpolarized) potential of the AHP.

In experiments examining I_T_, the peak current amplitude at each step potential (*V*) was first converted to conductance using the calculated reversal equilibrium potential of Ca^2+^ (*E*_Ca_) and *G* = I/(*E*_Ca_ − *V*), because driving force was linear over the range of voltages examined. The voltage dependencies of activation and steady-state inactivation were described with a single Boltzmann distribution, as follows: *G*(*V*) = *G*_max_/(1 − exp[(*V*_1/2_ − *V*_t_)/k]), where *G*_max_ is the maximal conductance, *V*_1/2_ is the half-maximal voltage, and *k* is the voltage dependence (slope) of the distribution. The current density of I_T_ at each tested membrane potential was determined by dividing the peak current by membrane capacitance.

To quantify the current density of I_NaP_, five sweeps of the current induced by the ramp protocol were averaged and smoothed with a 10 point boxcar filter. A linear fit from −78 to −70 mV was made to correct the leak current for each trace. TTX-sensitive sodium current was obtained by subtracting the averaged trace recorded under TTX from that under control conditions (Khaliq and Bean, 2010). The magnitude of I_NaP_ was measured at membrane potentials ranging from −70 to −40 mV at 2.5 mV intervals. Current density as a function of membrane potential was calculated by dividing the I_NaP_ determined at these intervals by membrane capacitance.

### Statistics

**Figure 1.**
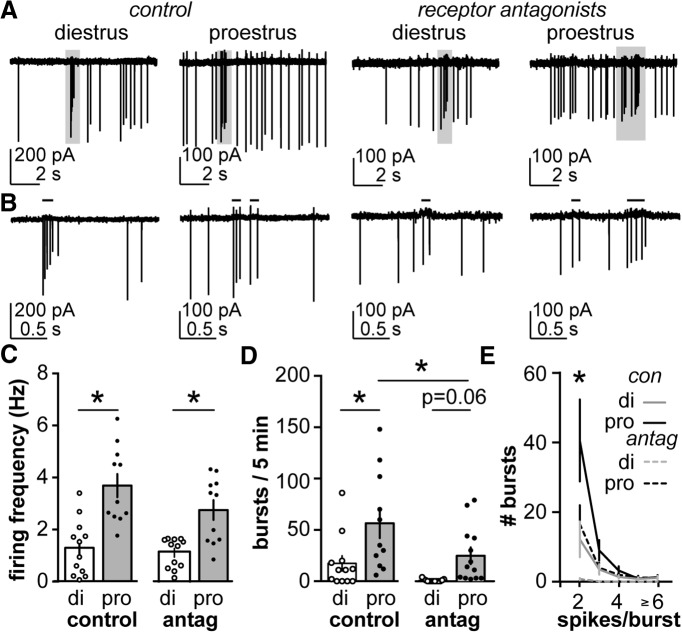
Firing frequency and burst firing in AVPV kisspeptin neurons are regulated by estrous cycle stage. ***A***, Representative extracellular recordings of AVPV kisspeptin neurons on di and pro under control conditions (left) and with AMPA, NMDA, and GABA_A_ receptors antagonized (right). ***B***, Areas in gray boxes expanded from ***A***. Black lines over traces in ***B*** indicate identified bursts. ***C***, ***D***, Mean ± SEM firing frequency (***C***) and number of burst events (***D***). ***E***, The number of burst events plotted as a function of number of spike per burst on diestrus (gray) and proestrus (black) under control conditions (solid line) and with receptor antagonists (dashed line). Antag, antagonists of ionotropic GABA and glutamate receptors. *p < 0.05 calculated by two-way ANOVA/Bonferroni or two-way RM ANOVA/Holm–Sidak test.

Data were analyzed using Prism 6 (GraphPad) and RStudio (RStudio, Inc.), and are reported as the mean ± SEM. The number of cells per group is indicated by *n*. Normality tests were performed using the test of D’Agostino and Pearson; all data were normally distributed except those in Figure 1*D* (two-way design) and Figure 7*C* (one-way design). All data requiring two-way analyses were compared using two-way ANOVA with Bonferroni *post hoc* analysis; this test is considered sufficiently robust for non-normally distributed data (Fig. 1*D* only; Underwood, 1997). Data requiring one-way analyses were compared using one-way ANOVA with Bonferroni *post hoc* analysis or Kruskal–Wallis (KW) test with Dunn’s *post hoc* analysis as dictated by data distribution (in only Fig. 7*C*, KW). For repeated measurements, two-way repeated-measures (RM) ANOVA with Holm–Sidak *post hoc* analysis was used. For paired data, a two-tailed paired Student’s *t* test was used. For categorical data analysis, a χ^2^ test of independence or Fisher’s exact test of independence was used to test the null hypothesis that categorical variables have no correlation with each other. An *F* test was used to test the null hypothesis that the SD for groups is equal. Linear regression was used to test the null hypothesis that slope is zero, and to measure the strength of the association [coefficient of determination (*r* ^2^)] between two variables. The null hypothesis was rejected if *p* < 0.05. *F*_DFn,DFd_ values from one-way ANOVA, two-way ANOVA, or two-way RM ANOVA are reported in Tables 1–3.

**Table 1:** Two-way ANOVA parameters for comparison among groups: cells from diestrus vs proestrus mice

Parameter (figure)	Estrous cycle stage	Antagonists	Interaction
Extracellular recordings			
Firing frequency (Fig. 1C)	*F*_(1,42)_ = 37.3***	*F*_(1,42)_ = 3.0	*F*_(1,42)_ = 1.2
Bursts/5min (Fig. 1D)	*F*_(1,46)_ = 14.8***	*F*_(1,46)_ = 8.4**	*F*_(1,46)_ = 0.8
Whole-cell recordings			
Input resistance (Fig. 2D)	*F*_(1,47)_ = 2.3	*F*_(1,47)_ = 7.5**a	*F*_(1,47)_ = 4.2*
Input resistance (Fig. 2C)	*F*_(1,37)_ = 1.7	*F*_(1,37)_ = 6.7*b	*F*_(1,37)_ = 1.2
Rebound: initial IF (Fig.2F)	*F*_(1,30)_ = 69.3***	*F*_(1,30)_ = 7.0*b	*F*_(1,30)_ = 80.3***
Rebound: overall frequency (Fig. 2G)	*F*_(1,34)_ = 34.8***	*F*_(1,34)_ = 7.3*b	*F*_(1,34)_ = 32.0***
First spike latency (Fig. 3B)	*F*_(1,37)_ = 0.8	*F*_(1,37)_ = 0.4b	*F*_(1,37)_ = 0.3
Rate of rise (Fig. 3C)	*F*_(1,37)_ = 0.6	*F*_(1,37)_ = 0.7b	*F*_(1,37)_ = 0.01
Threshold (Fig. 3D)	*F*_(1,37)_ = 0.1	*F*_(1,37)_ = 22.6***b	*F*_(1,37)_ = 3.8
FWHM (Fig. 3E)	*F*_(1,37)_ = 2.1	*F*_(1,37)_ = 2.8b	*F*_(1,37)_ = 0.2
AHP amplitude (Fig. 3F)	*F*_(1,37)_ = 0.7	*F*_(1,37)_ = 35.3***b	*F*_(1,37)_ = 0.1
AHP time (Fig. 3G)	*F*_(1,37)_ = 1.6	*F*_(1,37)_ = 26.2***b	*F*_(1,37)_ = 0.1

*^a^*Cesium antagonist.

*^b^*Tonic vs DIB.

*p < 0.05; **p < 0.01; ***p < 0.001.

**Table 2:** Two-way repeated-measures ANOVA for whole-cell comparison among groups: cells from diestrus vs proestrus mice

Parameter (figure)	Estrous cycle stage	Interaction	Matching
Control: spike/burst (Fig.1E)	*F*_(1,21)_ = 5.6*	*F*_(4,84)_ = 6.7***	*F*_(21,84)_ = 2.3**
Antagonists: spike/burst (Fig.1E)	*F*_(1,25)_ = 11.6**	*F*_(4,100)_ = 11.4***	*F*_(25,100)_ = 2.8***
I_T_ density (Fig. 4I)	*F*_(1,20)_ = 4.4*	*F*_(8,160)_ = 4.3***	*F*_(20,160)_ = 4.7***
I_NaP_ density (Fig. 5D)	*F*_(1,22)_ = 5.4*	*F*_(22,264)_ = 3.6***	*F*_(12,264)_ = 15.7***
Diestrus: tonic vs DIB (Fig. 2B)	*F*_(1,16)_ = 84.5***a	*F*_(6,96)_ = 82.6***	*F*_(16,96)_ = 2**
Proestrus: tonic vs DIB (Fig. 2B)	*F*_(1,17)_ = 42.2***a	*F*_(5,85)_ = 88.1*	*F*_(17,85)_ = 5.1***
Depolarize: IF (Fig. 4D)	*F*_(1,9)_ = 46.1***a	*F*_(2,18)_ = 52.2***	*F*_(9,18)_ = 11.2***
I_NaP_ density (Fig. 5E)	*F*_(1,16)_ = 7.3*b	*F*_(12,192)_ = 5.7***	*F*_(16,192)_ = 12.7***
I_NaP_ density (Fig. 5E)	*F*_(1,18)_ = 0.8c	*F*_(12,216)_ = 1.2	*F*_(18,216)_ = 17.8***
I_NaP_ density (Fig. 6D)	*F*_(1,10)_ = 3.5d	*F*_(12,120)_ = 2.3**	*F*_(10,120)_ = 10.2***

*^a^*Tonic vs DIB.

*^b^*Diestrus: rebound vs no rebound.

*^c^*Rebound: diestrus vs proestrus.

*^d^*4-AP sensitive vs insensitive.

*p < 0.05; **p < 0.01; ***p < 0.001.

**Table 3:** One-way ANOVA parameters for comparison among groups: OVX, OVX+E, and OVX+E+P

Parameter (figure)	Mice	Interaction	Matching
	OVX, OVX+E and OVX+E+P		
Extracellular recording			
Firing frequency (Fig. 7B)	*F*_(2,45)_ = 12.7***		
Whole-cell Recordings			
	OVX, OVX+E and OVX+E+P		
First spike latency (Fig. 7F)	*F*_(2,27)_ = 3.9*		
FWHM (Fig. 7G)	*F*_(2,27)_ = 6.3**		
AHP amplitude (Fig. 7H)	*F*_(2,27)_ = 7.6**		
Sag potential (Fig. 8F)	*F*_(2,27)_ = 35.9***		
Two-way repeated-measures ANOVA for comparison among groups: OVX and OVX+E			
	OVX vs OVX+E		
I_T_ (Fig. 8C)	*F*_(1,18)_ = 8.0*	*F*_(8,144)_ = 8.3***	*F*_(8,144)_ = 4.9***
I_NaP_ (Fig. 8E)	*F*_(1,19)_ = 5.8*	*F*_(12,228)_ = 4.3***	*F*_(19,228)_ = 12.5***
One-way ANOVA nonparameters KW test for comparison among groups: OVX, OVX+E, OVX+E+P			
	OVX, OVX+E and OVX+E+P		
Extracellular recording			
Bursts/5 min (Fig. 7C)	KW statistic 6.8		

*p < 0.05; **p < 0.01; ***p < 0.001.

## Results

### AVPV kisspeptin neurons exhibit higher spontaneous firing rates and more burst firing on proestrus than diestrus

The firing activity of GFP-identified kisspeptin neurons within the AVPV was monitored using targeted extracellular recordings in acutely prepared brain slices. All cells studied were spontaneously active during the 5 min observation period under both control conditions and after blocking ionotropic GABA_A_ and glutamate receptors. Figure 1, *A* and *B*, shows representative firing patterns from each group. Figure 1*C* shows the average firing frequency of AVPV kisspeptin neurons on diestrus (representing negative feedback) and proestrus (representing positive feedback) under control conditions (di, *n* = 12; pro, *n* = 11) or during antagonism of ionotropic receptors conveying GABAergic and glutamatergic fast synaptic transmission (di, *n* = 12; pro, *n* = 11). Consistent with a potential role in relaying estradiol-positive feedback, the spontaneous firing rate of AVPV kisspeptin neurons was greater on the afternoon of proestrus than that of diestrus (Fig. 1*C*; two-way ANOVA with Bonferroni correction, *p* < 0.0001). Antagonism of fast synaptic transmission via NMDA, AMPA, and GABA_A_ receptors did not alter the firing rate during either cycle stage (*p* > 0.9). The cycle-dependent difference in firing frequency was maintained after blocking ionotropic receptors (*p* = 0.0019).

Extracellular recordings were also used to evaluate burst versus single-spike firing. Bursts were defined as action potentials occurring within 105 ms of each other with progressively decreased amplitude and an upshift of baseline (Fig. 1*B*, black lines above traces). AVPV kisspeptin neurons exhibit spontaneous burst firing during both diestrus (*n* = 12) and proestrus (*n* = 11), but the number of the burst events per 5 min was higher on proestrus (Fig. 1*D*, two-way ANOVA with Bonferroni correction, *p* = 0.005). The number of the burst events per 5 min was decreased on proestrus in the presence of ionotropic glutamate and GABA_A_ receptor antagonists (di, *n* = 14; pro, *n* = 13; *p* = 0.02) but was not changed on diestrus (*p* = 0.14). Although it appears that the increase in the number of bursts in 5 min on proestrus compared with diestrus was maintained when ionotropic receptors were blocked, the *p* value was just short of that accepted for significance (Fig. 1*D*, *p* = 0.06).

We next analyzed the numbers of spikes per burst as a function of cycle stage (Fig. 1*E*, two-way RM ANOVA with Holm–Sidak control: di, *n* = 12; pro, *n* = 11; receptor antagonist: di, *n* = 14; pro, *n* = 13). Under control conditions, there were more bursts consisting of two spikes on proestrus than diestrus (*p* < 0.0001). After the blocking of ionotropic glutamate and GABA_A_ receptors, the number of two-spike bursts was decreased during both cycle stages (di, *p* = 0.01; pro, *p* < 0.0001), but was still higher on proestrus than diestrus (*p* = 0.005). No difference in the number of bursts with three or more spikes was detected between cycle stages either with or without ionotropic receptor antagonists. Following addition of the receptor antagonists, however, no cell studied during diestrus (*n* = 13) fired bursts with more than two spikes. This is in contrast to control conditions, under which 7 of 12 cells studied on diestrus fired bursts of three or more spikes. Cells studied on proestrus, in contrast, fired bursts with three or more spikes under control conditions as well as when ionotropic receptors were blocked. These observations suggest that both intrinsic properties and fast synaptic transmission likely contribute to burst firing in AVPV kisspeptin neurons, and that the relative contributions may change with cycle stage. We thus focused our remaining studies on the intrinsic properties of these neurons.

### Depolarization induces two firing patterns: tonic firing and depolarization-induced bursts

**Figure 2.**
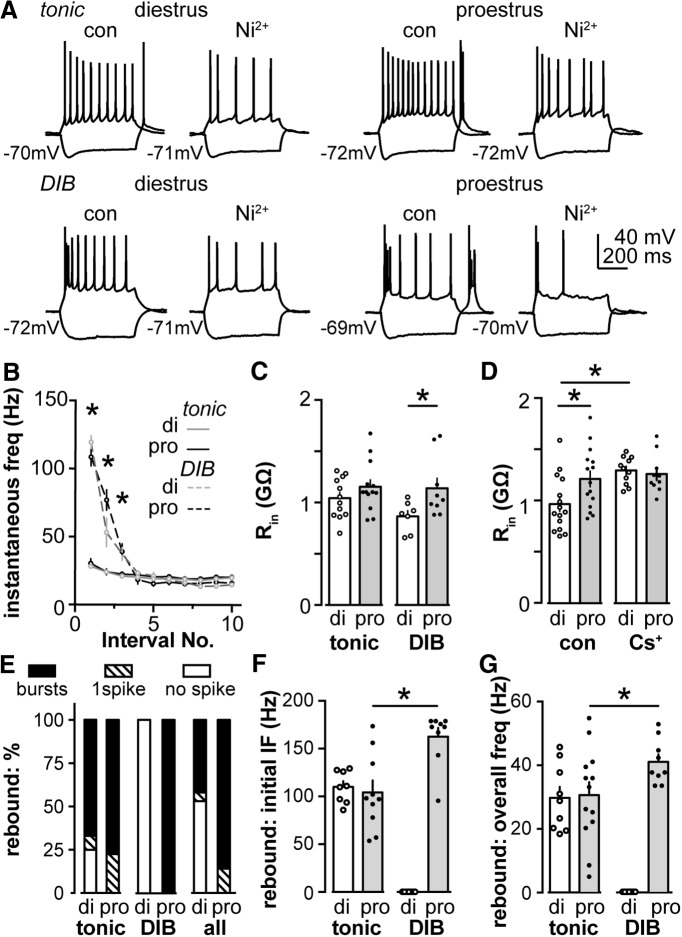
Depolarization and removal of hyperpolarization both induce distinct firing properties. ***A***, Representative firing properties of tonic and DIB cells on diestrus and proestrus under control conditions and during treatment with Ni^2+^ (100 µm). ***B***, Mean ± SEM IF of tonic (solid line) and DIB (dash line) cells on di (gray) and pro (black) plotted as a function of spike interval number. ***C***, R_in_ for tonic and DIB cells on diestrus and proestrus. ***D***, R_in_ for cells on diestrus and proestrus assessed using physiological (con) and Cs^+^-based pipette solution. ***E***, Distribution of cells that generated rebound bursts (black bar), one rebound spike (hatched bar), or no rebound spikes (white bar) on diestrus and proestrus for tonic, DIB, and all cells combined. ***F***, ***G***, Initial IF (***F***) and overall frequency (***G***) of rebound bursts in tonic and DIB cells on diestrus and proestrus. *p < 0.05 calculated by two-way ANOVA with Bonferroni correction or two-way RM ANOVA and Holm–Sidak test.

To begin to understand the contributions of the intrinsic properties of AVPV kisspeptin neurons to burst firing, we performed whole-cell current-clamp recordings on brain slices in the presence of APV, CNQX, and picrotoxin to antagonize effects attributable to the activation of ionotropic glutamate and GABA_A_ receptors. AVPV kisspeptin neurons exhibited two distinct firing patterns in response to depolarizing steady-state current injections of similar magnitude (25 ± 5 pA, 0.5 s) initiated from −70 ± 2 mV. Representative examples under control conditions are shown in Figure 2*A*. Tonic firing was defined as a steady firing rate with a consistent instantaneous frequency (IF; overall IF, 22 ± 1 Hz; initial IF, 29 ± 2 Hz vs final IF, 18 ± 1 Hz; paired *t* test, *p* < 0.0001; SD of IF, 4 ± 1 Hz; *n* = 25). Depolarization-induced bursting (DIB) cells exhibited an initial burst containing three to four spikes followed by mild frequency accommodation (overall IF, 73 ± 3 Hz; initial IF, 113 ± 4 Hz vs final IF, 13 ± 1 Hz; paired *t* test, initial IF vs final IF, *p* < 0.0001; SD of IF, 36 ± 2 Hz; *n* = 16; *F* test, tonic vs DIB SD, *p* < 0.0001). Tonic and DIB firing patterns were observed to a similar extent on both cycle stages studied (tonic vs DIB: di, 64% vs 36%, *n* = 19; pro, 59% vs 41%, *n* = 22; Fisher’s exact test, *p* > 0.9). We averaged the instantaneous firing frequency of each cell type on each cycle stage and plotted this as a function of spike interval number (Fig. 2*B*; tonic: di, *n* = 12; pro, *n* = 13; DIB: di, *n* = 7; pro, *n* = 9). The initial IF of DIB cells was fourfold larger than that in tonic cells on both cycle stages (Fig. 2*B*, two-way RM ANOVA with Holm–Sidak test, di, *p* < 0.0001; pro, *p* < 0.0001). The frequency plots did not show any difference between cycle stages in tonic or DIB cells (*p* > 0.1).

**Figure 3.**
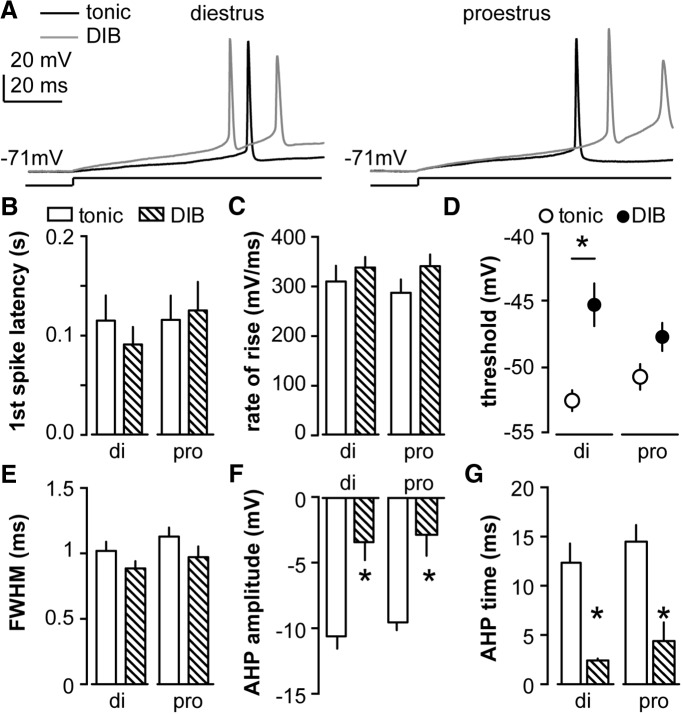
Action potential properties of AVPV kisspeptin neurons depend on firing pattern during depolarization but not cycle stage. ***A***, Representative first action potential evoked via minimal necessary depolarizing current in tonic (black) and DIB (gray) cells on diestrus (left) and proestrus (right). The line under the action potentials indicate timing of current injection (10 pA for examples shown except 15 pA for the DIB cell on proestrus). ***B****–****F***, Mean ± SEM action potential parameters in tonic cells (open bar for ***B***, ***C***, and ***E****–****G***; open circle for ***D***) and DIB cells (hatched bar for ***B***, ***C***, and ***E****–****G***; black circle for ***D***) on di and pro, including first spike latency (***B***), action potential rate of rise (***C***), threshold (***D***), FWHM (***E***), AHP potential amplitude (***F***), and AHP time on diestrus and proestrus (***G***). *p < 0.05 calculated by two-way ANOVA/Bonferroni test tonic vs DIB.

We next examined the action potential properties of tonic and DIB cells. In Figure 3*A*, the first action potential evoked by the minimum necessary depolarizing current injection is shown in tonic and DIB cells on diestrus (left) and proestrus (right). We measured and compared several action potential parameters between cycle stages and cell types (tonic: di, *n* = 12; pro, *n* = 13; DIB: di, *n* = 7; pro, *n* = 9). As summarized in Figure 3*B–G*, the action potential threshold, amplitude, and timing of the AHP potential showed a cell firing type-dependent but not cycle-dependent change (two-way ANOVA with Bonferroni correction, all *post hoc*, *p* < 0.001). The first spike latency, rate of rise, and FWHM of the action potential did not change among groups (*p* > 0.1 for all *post hoc* tests). The minimal necessary current injection (rheobase) itself was not different between cycle stages or cell types (di: tonic, 9.6 ± 1.0 pA; DIB, 13.3 ± 2.2 pA; pro: tonic, 10.8 ± 0.8 pA; DIB, 10.1 ± 1.1 pA; two-way ANOVA with Bonferroni correction, *p* > 0.1 for all comparisons).

There was no difference in the R_in_ of tonic firing cells on diestrus versus proestrus (Fig. 2*C*; two-way ANOVA with Bonferroni correction, *p* = 0.47; di, *n* = 12; pro, *n* = 13), but the R_in_ of DIB cells was greater on proestrus than diestrus (di, *n* = 7; pro, *n* = 9; *p* = 0.04). Within a cycle stage, there was no firing pattern-dependent (tonic vs DIB) difference in R_in_ (di, *p* = 0.91; pro, *p* = 0.22). The membrane capacitance was not different among groups (data not shown; *p* > 0.9). When grouped by cycle stage, cells recorded on proestrus had a greater R_in_ compared with those recorded on diestrus (di, *n* = 15; pro, *n* = 15; *p* = 0.01). This cycle-dependent difference in R_in_ was eliminated in recordings using a cesium-based pipette solution (Fig. 2*D*; *n* = 11 each; *p* = 0.88). Because R_in_ exhibited a cycle-dependent difference between diestrus and proestrus, and only DIB cells showed a cycle-dependent difference, it is likely DIB cells contribute the difference in R_in_ under control conditions. The elimination of a difference in R_in_ with Cs^+^ internal suggests a Cs^+^-sensitive potassium conductance under estrous cycle regulation may contribute to the difference in R_in_ values.

### Termination of hyperpolarization: rebound bursts and their relationship to the firing pattern during depolarization

In addition to depolarization-induced firing, many neurons exhibit firing upon termination of a hyperpolarizing stimulus (Perez-Reyes, 2003; Tadayonnejad et al., 2010). We tested whether AVPV kisspeptin neurons exhibit so-called rebound bursts. Cells were injected with hyperpolarizing current to achieve a membrane potential of −105 ± 3 mV. After the termination of hyperpolarization, most AVPV kisspeptin neurons exhibited rebound bursts (two or more spikes), whereas, the rest showed either single-rebound spikes or no rebound, as indicated in the representative examples in Figure 2*A* (control). The type of rebound events (bursts, single spike, or no spike) differed with cycle stage (Fig. 2*E*, right; di, *n* = 19; pro, *n* = 22; χ^2^ test, *p* < 0.001).

We next examined whether there was a relationship between the observed firing patterns during depolarization and the rebound patterns following the termination of hyperpolarization. Most cells (di, 8 of 12; pro, 10 of 13) that fired tonically upon depolarization had rebound bursts. The remaining cells studied on proestrus (3 of 13 cells) fired a single spike rebound, whereas most other cells studied on diestrus showed no rebound (3 of 12 cells), and one cell showed a single spike rebound. This distribution did not differ with cycle stage (Fig. 2*E*, left; χ^2^ test, *p* = 0.1). Strikingly, cells that fired DIB patterns had rebound bursts only on proestrus (Fig. 2*E*, middle; di, *n* = 7; pro, *n* = 10).

We focused on rebound bursts (two or more spikes) because they are more likely than single rebound spikes to achieve a sufficient change in intracellular Ca^2+^ to influence neurosecretion and thus postsynaptic events (Jacobs and Meyer, 1997). We characterized rebound bursts by measuring the initial IF (between spikes 1 and 2) and the overall frequency of rebound bursts (the number of spikes divided by the duration from the termination of hyperpolarization to the peak of the last rebound spike in the bursts). The initial IF and the overall frequency of bursts in tonic and DIB cells showed a firing pattern-dependent difference (Fig. 2*F*, initial IF; tonic: di, *n* = 8; pro, *n* = 10; DIB: di, *n* = 7; pro, *n* = 9; Fig. 2*G*, overall frequency; tonic: di, *n* = 9; pro, *n* = 13; DIB: di, *n* = 7; pro, *n* = 9; two-way ANOVA and Bonferroni correction). On proestrus, the initial IF and overall frequency was higher in DIB cells than tonic cells (IF, *p* < 0.0001; overall frequency, *p* = 0.03). There was no cycle-dependent change of rebound burst frequency in tonic cells (both initial and overall, *p* > 0.9), markedly different from DIB cells, which did not exhibit rebound bursts on diestrus. Our observations thus indicate that DIB and tonic cells show different responses to the termination of hyperpolarization. The different firing signatures may be linked to differences in specific ionic currents.

### Estrous cycle regulation of T-type calcium current properties and its role in depolarization and rebound firing patterns

**Figure 4.**
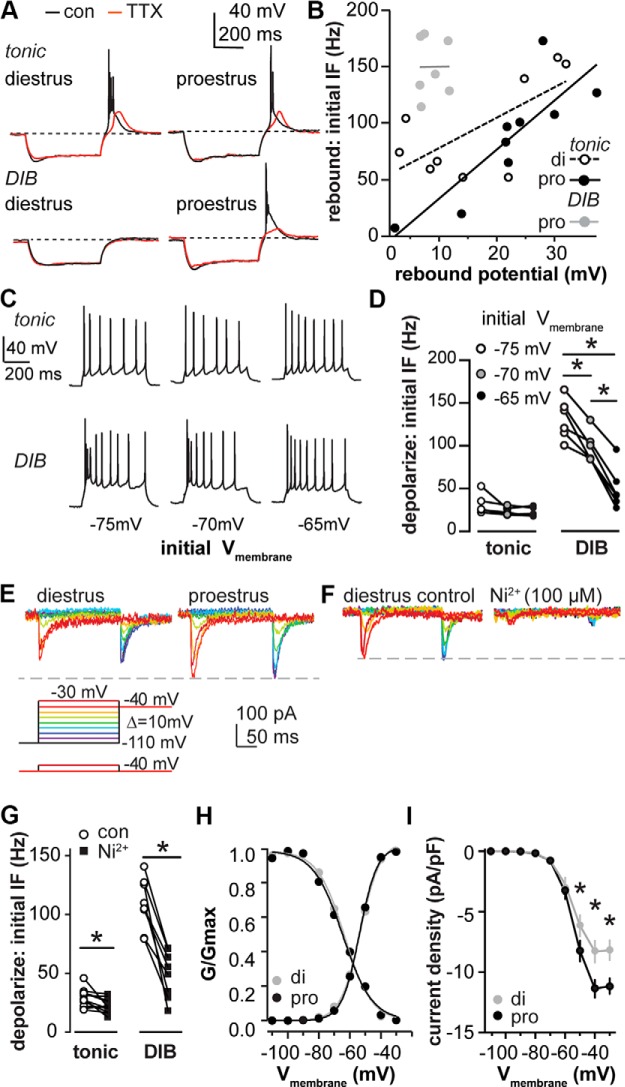
Ni^2+^-sensitive current is critical for bursting patterns and is under estrous cycle regulation. ***A***, Representative examples for rebound potential of tonic and DIB cells on diestrus and proestrus under control conditions (black) and then tested with TTX (1 µm) application (red). Dashed lines indicate −70 mV. ***B***, Positive correlation between rebound depolarization and initial IF was observed in tonic cells on di (open circle fitted with dashed line) and pro (black circle fitted with solid black line) but not in DIB cells on proestrus (gray circle fitted with gray line). ***C***, Representative examples of the depolarization-induced firing pattern of tonic and DIB cells initiated at −65, −70, and −75 mV. ***D***, IF was dependent on the preceding membrane potential in tonic and DIB cells, each line connects values from the same cells at different membrane potentials. ***E***, Voltage protocols for I_T_ isolation; bottom protocol was subtracted from the top to remove HVA contamination from step −30 mV (bottom, left). Representative isolated I_T_ on diestrus (top, left) and proestrus (top,right), each color represents a tested voltage. ***F***, Isolated I_T_ was blocked by Ni^2+^ (100 µm). ***G***, Initial IF of depolarization-induced firing for tonic and DIB cells under control conditions and with Ni^2+^ (100 µm) application, paired. ***H***, Activation and inactivation of I_T_ conductance was plotted and fit with Boltzmann function to derive *V*_1/2_ and *k* value on diestrus (gray dots fitted with gray line) and proestrus (black dots fitted with black line). ***I***, Mean ± SEM current density of I_T_ on diestrus and proestrus. *p < 0.05 calculated by two-way RM ANOVA and Holm−Sidak test.

The observation that cells with different depolarization-induced firing patterns could exhibit different rebound patterns based on cycle stage led us to examine potential underlying mechanisms in greater detail. We first tested the membrane response to the termination of hyperpolarization in the absence (to assess initial IF) and the presence of TTX to assess the depolarizing membrane response (Fig. 4*A*). After TTX application, marked membrane depolarization, often attributable to the activation of T-type calcium channels (Suzuki and Rogawski, 1989; Molineux et al., 2006), was observed following the termination of hyperpolarization in cells that fired rebound bursts but not in cells firing either single or no rebound spikes. This was not dependent on cycle stage but rather on the pre-TTX rebound profile. Figure 4*B* shows the correlation between the rebound IF and the amplitude of the depolarization. Rebound IF was positively correlated with rebound depolarization amplitude for tonic cells on both diestrus and proestrus (Fig. 4*B*; di: *r* ^2^ = 0.47; *p* = 0.045; *n* = 9; pro: *r* ^2^ = 0.84; *p* = 0.004; *n* = 9). In contrast, there was no correlation between these properties for DIB cells on proestrus (*n* = 7; *r* ^2^ < 0.001; *p* = 0.98).

The above observations suggest that, in addition to possible differences in currents driving action potential properties (Fig. 3*D*,*F*,*G*), other ionic currents might contribute to the difference between tonic firing and DIB cells. In other neuron types, the amplitude of rebound depolarization directly correlates with the size of transient Ca^2+^ currents (Suzuki and Rogawski, 1989; Molineux et al., 2006). We tested the effect of Ni^2+^ (100 µm, a dose that is fairly specific for T-type channels; Lee et al., 1999) on depolarization-induced firing patterns and hyperpolarization-induced rebound bursts. Ni^2+^ decreased the IF of the depolarization-induced bursts to approximately half (Fig. 2*A*, summary data; Fig. 4*G*, *n* = 8; paired *t* test, *p* = 0.0002). The initial IF after Ni^2+^ application was still higher in DIB cells than that in tonic cells under control (no Ni^2+^) conditions (*p* = 0.006). The initial IF of tonic cells also decreased after Ni^2+^ application (Fig. 4*G*; *n* = 11; paired *t* test, *p* = 0.049), but to a lesser extent (decreased 19 ± 7% in tonic vs 54 ± 6% in DIB cells; Student’s *t* test, *p* = 0.001). Since T-type channels are voltage dependent, we altered the initial membrane potential to −65, −70, or −75 mV, and then applied a current injection of 25 ± 5 pA to depolarize the membrane to generate *n* ± 2 action potential spikes (*n*, action potential numbers generated at −70 mV, with 25 ± 5 pA current injection; Fig. 4*C*). The IF of DIB cells was decreased because the initial membrane potential was depolarized from −75 to −70 to −65 mV (Fig. 4*D*; tonic, *n* = 5; DIB, *n* = 6; two-way RM ANOVA and Holm–Sidak test, all *post hoc* in DIB, *p* < 0.0001). No change in the IF of tonic cells was observed (*p* > 0.1). Rebound bursts were eliminated after Ni^2+^ application in the majority of cells recorded on both cycle stages (di, 7 of 8; pro, 6 of 7). The rebound bursts that Ni^2+^ did not block were reduced from three to four spikes per bursts to a single spike. Together, these observations suggest a substantial role for Ni^2+^-sensitive currents, perhaps *I*_T_, in both depolarization-induced and rebound firing patterns.

Persistence of single rebound spikes could be attributable to 100 µm Ni^2+^ not being sufficient to block all channels carrying *I*_T_. Alternatively, but not exclusively, other channels might affect the occurrence of the rebound bursts. We first measured T-type current properties in AVPV kisspeptin neurons. To facilitate the isolation of *I*_T_, a Cs^+^-based pipette solution was used to reduce potassium conductances, and TTX was bath applied to block fast voltage-dependent sodium channels. All cells recorded on diestrus and proestrus exhibited *I*_T_ based on the voltage dependence of the observed current. Figure 4*E* shows the representative whole-cell voltage-clamp traces of *I*_T_ triggered by the voltage protocol described in Materials and Methods. The application of 100 µm Ni^2+^ blocked a majority of the current (73.1 ± 0.4%; *n* = 4) evoked at a test pulse of −40 from −110 mV (Fig. 4*F*). This suggests that the currents observed are *I*_T_ and that most, but not all, of the current is sensitive to 100 µm Ni^2+^.

The conductance–voltage relationship was fit with a Boltzmann function. Neither the slope factor *k* nor the voltage dependence of activation or inactivation were different between cycle stages (Fig. 4*H*; *n* = 11 each; *V*_1/2_ activation: di, −54.5 ± 1.3 mV; pro, −53.5 ± 1.3 mV; *p* = 0.59; slope factor k: di, 5.1 ± 0.4; pro, 5.5 ± 0.4; *p* = 0.59; V_1/2_ inactivation: *n* = 12 each; di, −66.4 ± 1.6 mV; pro, −67.6 ± 1.5 mV; *p* = 0.56; slope factor k: di, −3.2 ± 0.1; pro, −3.0 ± 0.1; *p* = 0.11, Student’s *t* test). The current density, however, was greater on proestrus than diestrus at membrane potentials between −50 and −30 mV (Fig. 4*I*; *n* = 11; two-way RM ANOVA and Holm–Sidak test, *p* < 0.01). The change in current density was not attributable to a difference in either membrane capacitance or series resistance between groups.

### Other voltage-dependent currents modifying rebound bursts: persistent sodium current and A-type potassium current

The generation of rebound bursts can be a complex interplay of multiple channel types that pass currents in inward and outward directions. Since rebound firing is Ni^2+^ sensitive, I_T_ likely plays a dominant role in generating rebound bursts. The lower likelihood of firing rebound bursts on diestrus versus proestrus may be attributable to estrous cycle regulation of other currents that either facilitates or inhibits rebound burst generation. One candidate for a facilitating current is the I_NaP_, as it activates at membrane potentials in the subthreshold range.

**Figure 5.**
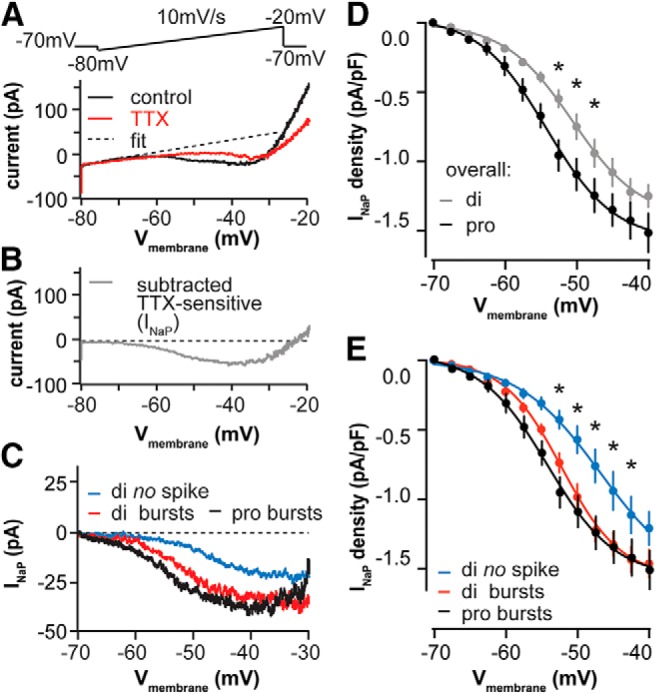
I_NaP_ facilitates rebound burst generation and is regulated by the estrous cycle. ***A***, ramp protocol (top) and representative raw currents (bottom) under control conditions (black) and after application of TTX (red). Dashed line indicates the linear fit to correct for leak current. ***B***, Representative TTX-sensitive I_NaP_ obtained by subtracting the TTX trace from the control trace in ***A*** after linear fit of each. ***C***, Representative I_NaP_ in cells that exhibit rebound bursts (red) or no rebound spikes (blue) on diestrus and those that exhibited rebound bursts on proestrus (black). ***D***, ***E***, Mean ± SEM I_NaP_ current density on di vs pro (***D***) and with cells parsed by cycle stage and rebound firing (***E***). *p < 0.05 calculated by two-way RM ANOVA and Holm–Sidak test.

We characterized I_NaP_ in AVPV kisspeptin neurons on diestrus and proestrus using slow (10 mV/s) voltage ramps from −80 to −20 mV (Fig. 5*A*, top). The ramp-induced current was linear from −80 mV to approximately −65 mV (Fig. 5*A*, bottom). At membrane potentials more depolarized than −65 mV, we observed a persistent inward current that peaked near −40 mV. This current was blocked by TTX, suggesting the net current is I_NaP_ (Fig. 5*B*). The current density of I_NaP_ was higher on proestrus than diestrus from −55 to −47.5 mV (Fig. 5*C*,*D*; *n* = 12 each; two-way RM ANOVA and Holm–Sidak test: −55 mV, *p* = 0.057; −52.5 mV, *p* = 0.002; −50 mV, *p* = 0.01; and −47.5 mV, *p* = 0.045).

Because I_NaP_ appears to be regulated by the estrous cycle, we tested whether it facilitates rebound burst generation. We divided cells on diestrus into those exhibiting rebound bursts (two or more spikes) versus those not exhibiting any rebound spikes, and compared the I_NaP_ current density of these two groups with that of cells on proestrus (all of which had rebound bursts with two or more spikes). Figure 5*C* shows a representative I_NaP_ for each group, and Figure 5*E* shows the quantitative current density comparison among groups. I_NaP_ current density was lower in cells on diestrus that do not fire rebound bursts (*n* = 11) than in those that fire rebound bursts (*n* = 9) at membrane potentials between −52.5 and −42.5 mV (two-way RM ANOVA and Holm–Sidak test, *p* < 0.05). I_NaP_ current density between cells exhibiting rebound bursts on diestrus (*n* = 9) and proestrus (*n* = 11) was not different (*p* > 0.6). This suggests that cells that do not fire rebound spikes likely account for the lower I_NaP_ current density on diestrus versus proestrus.

**Figure 6.**
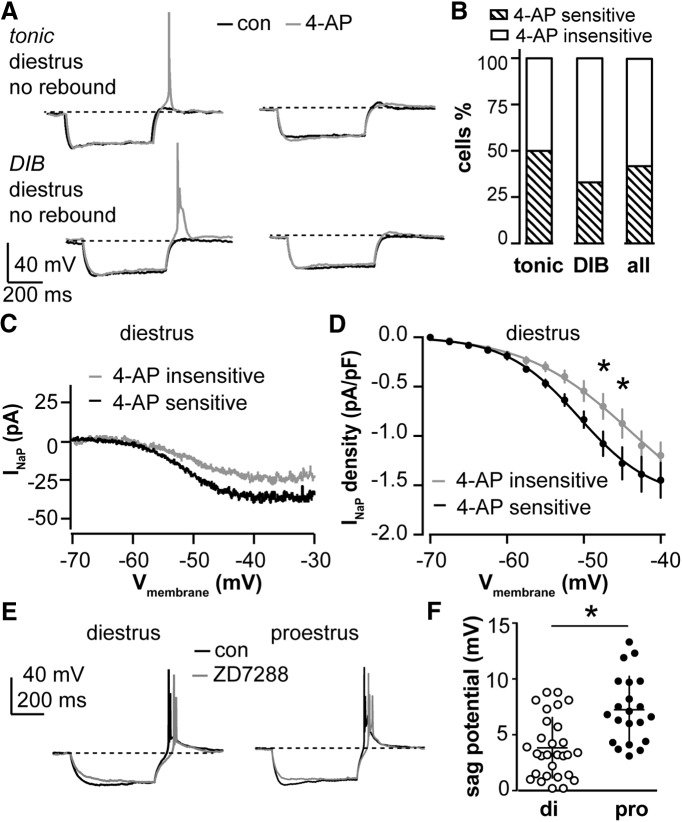
Subpopulations of cells that exhibit no rebound spikes on diestrus have restored ability to generate rebound spikes after blocking I_A_. ***A***, Representative firing properties of tonic and DIB cells on diestrus that did not exhibit rebound spikes under control conditions (black): 4-AP (5 mm) treatment (gray) restored rebound spikes in some cells (left) but not others (right). ***B***, Percentage of cells with 4-AP-sensitive (hatched bar) or 4-AP-insensitive rebound firing (open bar) for tonic, DIB, and all cells combined on diestrus. ***C***, Representative I_NaP_ in 4-AP-sensitive (black) and 4-AP-insensitive (gray) cells tested on diestrus. ***D***, Mean ± SEM I_NaP_ current density for 4-AP-sensitive (black) and 4-AP-insensitive (gray) cells tested on diestrus. *p < 0.05 calculated by two-way RM ANOVA and Holm–Sidak test. ***E***, Representative cells on diestrus and proestrus preserved rebound bursts but not sag potential after ZD7288 (50 µm) application. ***F***, Sag potential was increased on proestrus compared to diestrus. *p < 0.05, Student’s *t* test.

We also considered the possibility that an outward current counteracts inward current from *I*_NaP_ and *I*_T_ to decrease the burst occurrence on diestrus. Because Cs^+^-sensitive potassium channels likely contribute to the decreased R_in_ on diestrus versus proestrus, we hypothesized that potassium currents may contribute to the silencing of bursts on diestrus for both tonic and DIB cells. In particular, we examined the 4-AP-sensitive *I*_A_ because it can be activated at relatively hyperpolarized potentials and is thus more likely to play a role in modifying spike initiation (Perreault and Avoli, 1991; DeFazio and Moenter, 2002; Amendola et al., 2012). We performed current-clamp recordings to identify the tonic or DIB cells that did not exhibit rebound spikes on diestrus, then treated these cells with 4-AP (5 mm). Of the 12 cells we tested, 5 exhibited rebound during 4-AP treatment (4 cells had one rebound spike and 1 cell had a two-spike burst; Fig. 6*B*, right). These were referred to as 4-AP-sensitive cells. The remaining seven cells did not initiate spikes upon rebound following 4-AP treatment (4-AP insensitive). Figure 6*A* shows the representative tonic and DIB cells that were 4-AP sensitive (left) and insensitive (right). Figure 6*B* shows that half of the tonic cells (Fig. 6*B*, left; *n* = 6) and one-third of DIB cells (Fig. 6*B*, middle; *n* = 6) were 4-AP sensitive. We then compared I_NaP_ in cells that were 4-AP sensitive (*n* = 5) and insensitive (*n* = 7), and found that current density was greater in the 4-AP-sensitive group (Fig. 6*C*,*D*; two-way RM ANOVA and Holm–Sidak test: −47.5 mV, *p* = 0.04; −45 mV, *p* = 0.02). The R_in_ of the tested cells increased after 4-AP treatment, which is consistent with the above change when a Cs^+^-based pipette solution was used (paired *t* test, *n* = 11: control vs 4-AP, 858 ± 72 vs 1044 ± 98 MΩ, *p* = 0.005). This suggests that, for a subset of neurons, potassium channels may play an active role in preventing burst generation that may be independent of the regulation of I_NaP_ and I_T_.

Some AVPV kisspeptin neurons are known to express hyperpolarization-activated nonselective cation channels (HCN) and exhibit a sag in membrane potential during current clamp typical of cells exhibiting hyperpolarization-activated current (*I*_h_; Piet et al., 2013). We observed that after achieving a hyperpolarized membrane potential of −105 ± 3 mV, 70% of AVPV kisspeptin neurons showed sag potential (>2 mV) on diestrus (*n* = 29; 3.8 ± 0.5 mV), and 95% on proestrus (*n* = 20; 7.3 ± 0.7 mV). Our results confirmed that the sag potential is cycle dependent (Fig. 6*F*; Student’s *t* test, *p* = 0.0002). To examine the role of I_h_ in generating rebound bursts, we blocked HCN channels using ZD7288 (50 µm; Fig. 6*E*). This eliminated the sag potential but did not affect the number of spikes per rebound burst for cells tested on either stage (control: 3.1 ± 0.2 spikes/burst; ZD7288, 2.9 ± 0.2 spikes/burst; *p* = 0.17, paired Student’s *t* test; *n* = 9 total, five diestrus, four proestrus), which is consistent with previous findings (Zhang et al., 2013b). This suggests that although I_h_ is regulated by the estrous cycle, it may play a small role or no role in generating rebound bursts under our experimental conditions.

### Estrous cycle regulation of burst properties is attributable to circulating estradiol, not progesterone

The cycle-dependent changes in burst properties observed above are most likely attributed to estrous cycle-dependent changes in circulating levels of ovarian sex steroids, in particular estradiol and/or progesterone. To determine the role of specific gonadal steroids in these biophysical properties, we set up the following four groups of mice: OVX, OVX+E, OVX+E+P, and OVX+E+V to rule out the potential effects induced by injection-associated stress in OVX+E+P mice. OVX mice treated with P only were not tested because a single-cell real-time PCR scan (as in Ruka et al., 2013) of AVPV kisspeptin neurons from OVX mice indicated only 2 of 10 cells expressed the estrogen-dependent progesterone receptor mRNA three days after ovariectomy, compared to 7 of 9 cells from OVX mice treated with estradiol (not shown).

**Figure 7.**
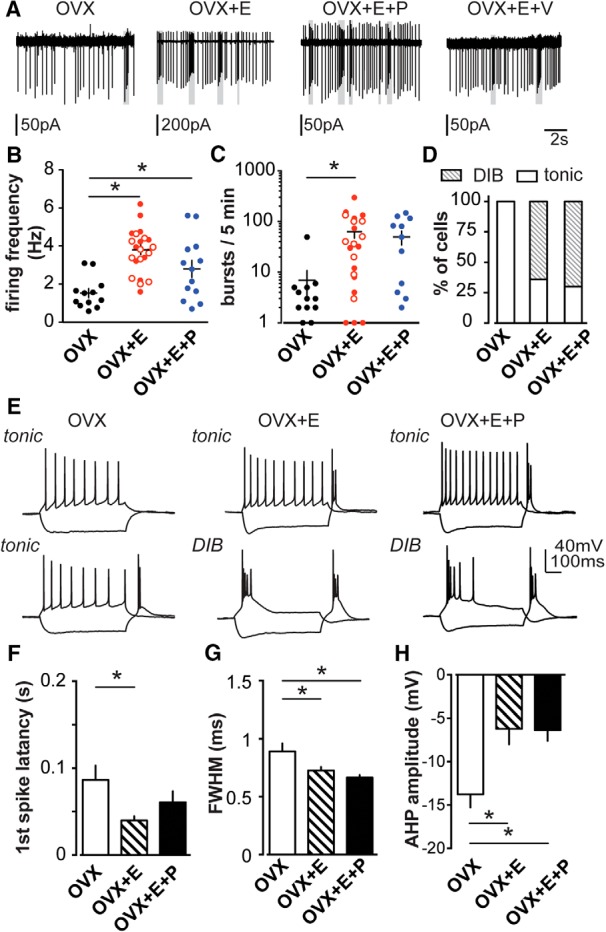
Estradiol but not progesterone increases overall excitability and burst events in AVPV kisspeptin neurons. ***A***, Representative extracellular recordings of OVX, OVX+E, OVX+E+P, and OVX+E+V with AMPA, NMDA, and GABA_A_ receptors antagonized. Gray boxes indicate bursts. ***B***, ***C***, Mean ± SEM firing frequency (***B***) and number of burst events (***C***) of OVX (black circles), OVX+E (solid red circles), OVX+E+V (open red circles), and OVX+E+P (blue). ***D***, Percentage of cells that fire DIB or tonic patterns on OVX, OVX+E, and OVX+E+P groups. ***E***, Representative firing properties of tonic (top) and DIB (bottom; except OVX) cells in OVX, OVX+E, and OVX+E+P groups. ***F–H***, Mean ± SEM of action potential parameters in OVX (white), OVX+E (hatched), and OVX+E+P (black) groups: latency to first spike (***F***), action potential FWHM (***G***), and AHP potential (amplitude; ***H***). *p < 0.05 calculated by one-way ANOVA/Bonferroni test or Kruskal–Wallis or Dunn’s test, as dictated by data distribution.

Short-term firing patterns of AVPV kisspeptin neurons were monitored (Fig. 1) in all four groups of mice (OVX, OVX+E, OVX+E+V, and OVX+E+P) via extracellular recordings with antagonized AMPA, NMDA, and GABA_A_ receptors. There were no differences in firing rate or bursts between OVX+E and OVX+E+V mice when we compared all four groups (one-way ANOVA with Bonferroni correction, *p* > 0.99), indicating that injection alone causes no detectable change in the firing properties in AVPV kisspeptin neurons. OVX+E and OVX+E+V cells were thus combined for burst analyses and are reported as OVX+E in Figure 7, *B* and *C*; vehicle-treated animals were not included in further studies. Firing frequency in OVX+E and OVX+E+P cells was increased compared with OVX cells (*p* < 0.0001 and *p* < 0.02, respectively), whereas no difference was observed between OVX+E+P and OVX+E cells (Fig. 7*A*,*B*; one-way ANOVA with Bonferroni correction). Spontaneous bursting events were increased in cells from OVX+E and OVX mice (Fig. 7*C*; one-way ANOVA with KW/Dunn’s test, *p* = 0.03). The number of bursts in cells from OVX+E+P mice was intermediate to and not different from that in cells from either OVX or OVX+E mice. These observations suggest that estradiol alters the firing frequency and pattern of AVPV kisspeptin neurons, and that the addition of progesterone does not appear to further shift these parameters.

To examine steroid effects on action potential properties, whole-cell recordings were used to capture the firing signature of AVPV kisspeptin neurons in OVX, OVX+E, and OVX+E+P groups in response to the depolarization and removal of hyperpolarization (Fig. 7*E*). Most cells from OVX+E mice (64%, *n* = 7 of 11) and OVX+E+P mice (67%, 6 of 9) exhibit DIBs. In contrast, DIBs were not observed in cells from OVX mice, and all cells from these mice fired in a tonic manner upon depolarization (Fig. 7*D*; *n* = 10; χ^2^ test, *p* = 0.003). In response to the removal of hyperpolarization, more cells fire rebound bursts in the OVX+E and OVX+E+P groups than in the OVX group: only 30% of OVX cells (3 of 10) fire rebound bursts, whereas ∼90% of OVX+E cells (10 of 11) and OVX+E+P cells (8 of 9) fire rebound bursts (χ^2^ test, *p* = 0.003). The number of spikes in rebound bursts was increased in cells from OVX+E mice (4.7 ± 0.9, *n* = 11) compared with OVX (0.9 ± 0.3, *n* = 10; one-way ANOVA with Bonferroni correction, OVX vs OVX+E mice, *p* = 0.0007). This parameter had an intermediate value in cells from OVX+E+P mice (2.9 ± 0.5; *n* = 9). The latency to the first spike was decreased in cells from OVX+E versus OVX mice (*p* = 0.02); again this parameter had an intermediate value in cells from OVX+E+P mice (Fig. 7*F*). FWHM and AHP amplitude were increased in cells from both OVX+E and OVX+E+P mice compared with those observed in OVX mice (Fig. 7*G*,*H*; Table 3).

**Figure 8.**
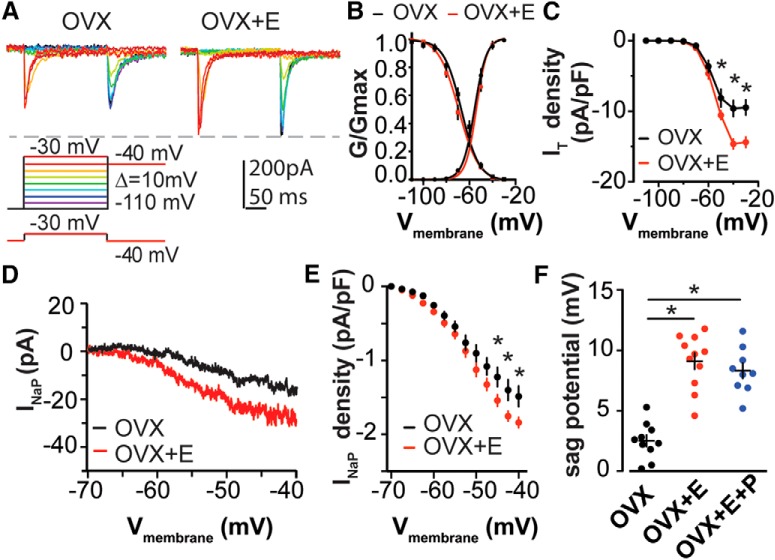
Estradiol increases *I*_T_ and *I*_NaP_ density in AVPV kisspeptin neurons. ***A***, Voltage protocol (bottom) and representative subtraction-isolated *I*_T_ in OVX (top left) and OVX+E groups (top right). ***B***, Activation and inactivation of *I*_T_ conductance was plotted and fit with a Boltzmann function to derive *V*_1/2_ and *k* value in OVX (black) and OVX+E (red). ***C***, Mean ± SEM current density of *I*_T_ in OVX (black) and OVX+E (red). ***D***, Representative *I*_NaP_ in OVX (black) and OVX+E (red) groups. ***E***, Mean ± SEM *I*_NaP_ density in OVX (black) vs OVX+E (red) groups. ***F***, Mean ± SEM of sag potential induced by hyperpolarization. *p < 0.05 calculated by two-way RM ANOVA/Holm-Sidak test for ***D*** and ***E***; *p < 0.05 calculated by one-way ANOVA/Bonferroni test for ***F***.

We also examined the effects of estradiol on the modulation of I_T_ and I_NaP_, as detailed above. Estradiol increased *I*_T_ current density (Fig. 8*A*,*C*; OVX, *n* = 10; OVX+E, *n* = 10; two-way RM ANOVA and Holm–Sidak test: −50 mV, *p* = 0.04; −40 mV, *p* < 0.001; −30 mV, *p* < 0.001) as well as I_NaP_ current density (Fig. 8*D*,*E*; OVX, *n* = 10; OVX+E, *n* = 11; two-way RM ANOVA and Holm–Sidak test: −50 mV, *p* = 0.02; −45 mV, *p* = 0.01; −40 mV, *p* = 0.01). Similar to the lack of change in voltage-dependent activation and inactivation of I_T_ between diestrous and proestrous phases of the estrous cycle, estradiol did not affect these parameters (Fig. 8*B*; activation, *n* = 10 each: *V*_1/2_: OVX, −55.3 ± 1.2; OVX+E, −54.0 ± 1.0; *p* = 0.41; slope factor *k*: OVX, 5.6 ± 0.5; OVX+E, 5.3 ± 0.4; *p* = 0.51; inactivation, *n* = 10 each: *V*_1/2_: OVX, −66.3 ± 1.3; OVX+E, −69.1 ± 1.5; *p* = 0.09; slope factor k: OVX, −3.3 ± 0.2; OVX+E, −3.3 ± 0.2; *p* = 0.80, Student’s *t* test). The sag potential, which represents the activation of the HCN channel at the hyperpolarized potential, was increased in cells in the OVX+E and OVX+E+P groups compared with that observed in the OVX group (Fig. 8*F*; one-way ANOVA with Bonferroni correction, OVX+E vs OVX and OVX+E+P vs OVX, *p* < 0.0001). These observations suggest that most cycle-dependent effects on AVPV kisspeptin neuron firing and associated currents are attributable to estradiol.

## Discussion

**Figure 9.**
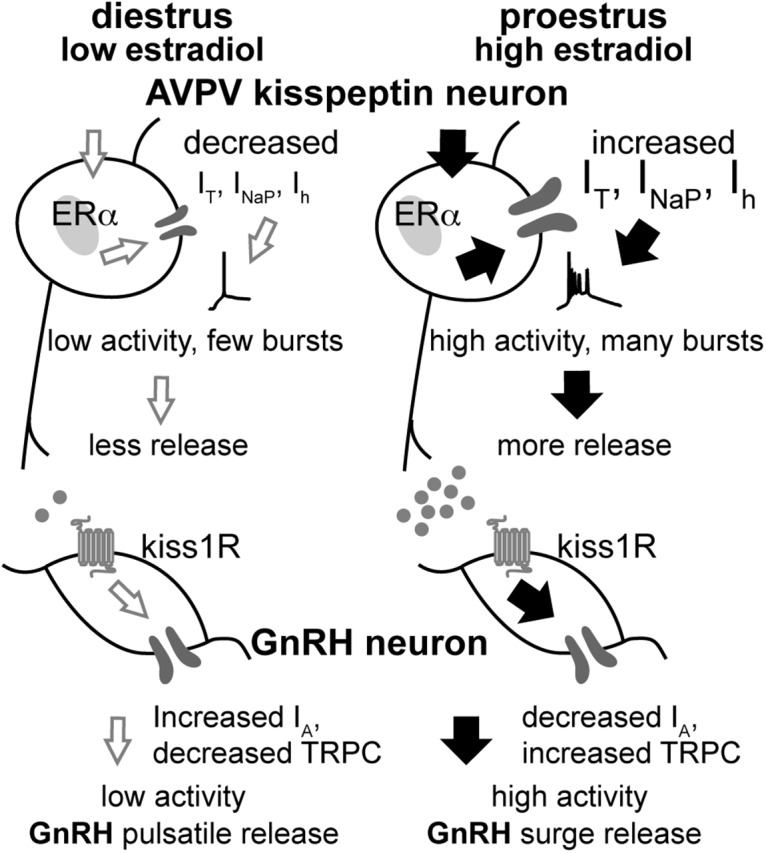
Schematic model of cyclic regulation of the AVPV kisspeptin–GnRH circuitry. On diestrus, low estradiol levels exert negative feedback and AVPV kisspeptin neurons exhibit low I_T,_ I_NaP_, and I_h_. This contributes to reduced overall and burst firing, thus lower release of kisspeptin, GABA, and glutamate. GnRH neurons exhibit low activity and a pulsatile release pattern. On proestrus, high levels of estradiol exert positive feedback action, increasing *I*_T,_ *I*_NaP_, and *I*_h_, and facilitate increased overall excitability and, for the former two currents, increase burst firing. This increases neurosecretion from these neurons, increasing excitatory drive to GnRH neurons via kisspeptin, GABA, and glutamate, and also increasing GnRH neuron activity via kisspeptin-mediated reduction of I_A_ (Pielecka-Fortuna et al., 2011) and increase in TRPC (transient receptor potential canonical) conductance (Zhang et al., 2013a). Together, the intrinsic changes and increased excitatory drive provoke increased release of GnRH (Glanowska et al., 2012).

Steroid milieu and other cues are integrated to generate a GnRH release pattern as the central output signal controlling fertility. In most vertebrates, estradiol-positive feedback induces a surge of GnRH release that is crucial for ovulation (Moenter et al., 1992; Christian and Moenter, 2010). GnRH neurons receive estradiol feedback mainly via steroid-sensitive afferents, including AVPV kisspeptin neurons (Pinilla et al., 2012). We demonstrated AVPV kisspeptin neurons increase overall and burst firing rate on proestrus versus diestrus, and revealed the estrous cycle regulates the interactions of multiple conductances contributing to burst generation primarily via cycle-dependent changes in circulating estradiol levels (Fig. 9).

Burst firing is implicated in the increasing reliability of neural information processing, synaptic plasticity, and neuropeptide/neuroendocrine secretion (Krahe and Gabbiani, 2004; van den Pol, 2012). For the latter, increased cytoplasmic calcium induced by bursts may enhance dense-core vesicle fusion with the plasma membrane (Jackson et al., 1991; Muschol and Salzberg, 2000). Shifts toward increased AVPV kisspeptin neuron firing frequency and burst events may thus indicate increased neurosecretion.

All AVPV kisspeptin neurons examined were spontaneously active, but activity was greater on proestrus. The increased activity of AVPV kisspeptin neurons from proestrous and OVX+E mice is consistent with elevated cFos expression in this cell population during the preovulatory or estradiol-induced LH surge (Smith et al., 2006; Clarkson et al., 2008). Of note, other studies (Ducret et al., 2010; De Croft et al., 2012; Piet et al., 2015) found no cycle-dependent shift in the spontaneous firing rate of AVPV kisspeptin neurons, with reports of trends toward either increased or decreased activity on proestrus. This contrast may in part be attributable to methodological differences, including slice thickness, recording duration, and the time of day of the experiment. Activity of the reproductive neuroendocrine circuitry, particularly that involved in estradiol-positive feedback, is regulated by the time of day (Christian and Moenter, 2010). Slices in the present study were prepared at zeitgeber time 10 (ZT10) to ZT11, at the peak of the reported increase in the expression of cFos in AVPV kisspeptin neurons in OVX+E mice (ZT9 to ZT12; Robertson et al., 2009; Kriegsfeld, 2013), whereas previous studies were conducted from ZT4 to ZT6. Notably, the firing frequency of AVPV kisspeptin neurons in the present study increased by ∼20-30% after an 8–10 min stable recording, limiting confidence in the analysis of longer-term firing patterns.

Burst firing may be attributable to intrinsic mechanisms and/or synaptic inputs. AVPV kisspeptin neurons receive glutamate and GABA synaptic inputs (Frazão et al., 2013), the latter of which is known to be regulated by both time of day and estradiol (DeFazio et al., 2014). Blocking fast synaptic transmission reduced, but did not eliminate, burst firing in the present study, suggesting that intrinsic mechanisms contribute to bursts. Although a role for other transmitters/peptides in AVPV kisspeptin neuron burst generation cannot be eliminated, we focused on the intrinsic ionic conductances underlying firing and burst generation.

AVPV kisspeptin neurons were classified as tonic or DIB neurons based on firing patterns evoked by depolarizing these cells from their baseline membrane potential. Both tonic and DIB cells exhibited rebound bursts induced by hyperpolarization termination. The percentage of cells exhibiting DIB and rebound bursts was increased by estradiol (OVX vs OVX+E mice), with rebound bursts also increasing on proestrus versus diestrus. Importantly, we observed cells in slices from the same diestrous mouse that both fire and fail to exhibit rebound bursts. This suggests that these differences are unlikely to be attributed to different steroid levels *in vivo*. One postulate is that the difference in rebound burst firing in cells on diestrus is related to the expression of ERα, which may affect the ionic conductance profiles and firing patterns. Of interest, the percentage of AVPV kisspeptin neurons expressing ERα is ∼65% (Clarkson et al., 2008; Mayer et al., 2010) versus ∼60% of cells on diestrus exhibiting no rebound burst properties. This increase in elicited bursts was consistent with increased spontaneous firing during both experimentally induced or cycle-dependent elevation of estradiol. Together, these studies suggest depolarization and release from hyperpolarization as two possible mechanisms for generating the spontaneous bursts observed in extracellular recordings. DIB was not observed in the absence of ovarian-derived estradiol, indicating a critical role for this steroid in generating closely spaced spikes upon depolarization. Our results add new, functional parameters to classify AVPV kisspeptin neurons. Future studies will focus on combining the electrophysiological properties with molecular signatures to uncover the specific roles that each cellular subtype may perform.

The distinct action potential signatures of tonic and DIB patterns suggest that different channel types may be contributing to action potential firing in these cells (Bean, 2007). We next began to decipher the ionic conductances underlying firing properties in tonic and DIB cells. The presence of currents at subthreshold membrane potentials can enhance or retard spike generation to influence the overall firing patterns in many brain regions (DeFazio and Moenter, 2002; Puopolo et al., 2007; Chu et al., 2010; Itri et al., 2010; Khaliq and Bean, 2010), including AVPV kisspeptin neurons (Zhang et al., 2013b, 2015). In the present study, the sensitivity of depolarization-induced bursting to Ni^2+^ and initial membrane potential further suggests that this firing pattern may arise from conductances that are voltage dependent and/or Ni^2+^ sensitive. Rebound bursts were also Ni^2+^ sensitive. Further, the positive correlation between rebound depolarization when firing was blocked and the instantaneous frequency of rebound bursts further suggests that I_T_ may also be important to rebound burst generation. Of note, no such correlation was observed in DIB cells; this may be attributed to a higher potassium conductance near the baseline potential opposing the action of I_T_.

Because Ni^2+^ is not a complete or exclusive blocker of I_T_, and it is difficult to attribute the role of a specific conductance to observations in current-clamp recordings, voltage clamp was used to isolate I_T_. The biophysical parameters of I_T_ in AVPV kisspeptin neurons in the present study are consistent with the parameters measured in other neuronal population (Molineux et al., 2005; Talavera and Nilius, 2006; Nelson et al., 2007). The increased current density on proestrus and in cells from OVX+E mice in the absence of shifts in voltage dependence suggests that an increased number of channels in the membrane may contribute to increased bursting. Interestingly, I_T_ current density differed at physiologically relevant membrane potentials critical for spike generation. Questions remain about whether tonic and DIB cells have distinct I_T_ current densities because firing properties are difficult to determine with the Cs^+^-based pipette solution used to quantify I_T_.

Interestingly, all the cells tested on diestrus exhibited I_T_, raising the question of why only half of these cells exhibit rebound bursts. The latter may be attributable to a decreased depolarizing conductance and/or an increased hyperpolarizing conductance in cells without rebound bursts. We targeted I_NaP_, I_h_, and I_A_ to test this postulate because of their activation at subthreshold membrane potentials. Previous studies show that I_NaP_ density in AVPV kisspeptin neurons was increased in mice treated with high estradiol versus low estradiol levels (Zhang et al., 2015). We found that both I_NaP_ and I_A_ currents may sculpt burst firing in AVPV kisspeptin neurons. The lack of specific I_NaP_ blockers precludes parsing its role compared with that of I_T_ in generating rebound bursts, but the upregulation of I_NaP_ upon specific manipulation of estradiol suggests that this steroid plays a role in the cycle-dependent changes observed. Cells that did not fire rebound bursts had decreased I_NaP_ relative to cells that fired rebound bursts in diestrous mice. These observations suggest that interactions among various currents alter burst firing. Blocking I_A_ permitted rebound spikes in half of the cells recorded on diestrus regardless of their depolarization-evoked firing pattern. Interestingly, I_NaP_ was also different between 4-AP-sensitive and 4-AP-insensitive cells near the action potential threshold, suggesting that the increased persistent sodium current in the 4-AP-sensitive group may be able to influence burst firing with the removal of I_A_. In the 4-AP-insensitive group, the removal of I_A_ did not lead to rebound bursts, suggesting that a relatively small I_T_ and/or I_NaP_ is not able to evoke rebound spikes. In the present studies, blocking the I_h_ did not affect burst firing induced by current injections during the reproductive cycle. This current may still play a role in spontaneous bursting in these neurons via interactions with other conductances activated at subthreshold potentials. In this regard, other studies have not found an effect of blocking *I*_h_ on firing rate during proestrus, although an increase in spike latency was observed (Piet et al., 2013; Zhang et al., 2013b). Our results suggest an interaction among two transient currents (I_T_ and I_A_) and one persistent current (I_NaP_) that may modulate the bursting generation in AVPV kisspeptin neuronal population, supporting and extending previous work (Zhang et al., 2015). Furthermore, potassium currents also appear to play a role in regulating passive membrane properties by increasing the input resistance on proestrus compared to diestrus. This may result in an increased gain of the cells to respond to their afferent stimulus.

These observations are consistent with the increasing understanding through experimental and computational studies that different interactions among ionic conductances can generate the same firing properties in many neurons across species (Goldman et al., 2001; Puopolo et al., 2007). Neurons can achieve similar firing output by adding, deleting, or modulating different conductances (Prinz et al., 2004; Swensen and Bean, 2005). In AVPV kisspeptin neurons, the present results indicate that bursts can be generated via depolarization or release of hyperpolarization, both of which may recruit multiple ionic conductances. Bursts may be triggered and facilitated by increased I_T_, I_NaP_, or decreased I_A_; and they may be silenced or reduced (e.g., number of events and frequency) with a combination of decreased I_T_ and I_NaP_, and increased I_A_. In a cell type that is likely critical to reproduction, the ability to produce the appropriate output via a variety of scenarios is pertinent to the survival of the species.

Another question is which cycle-dependent cues are responsible for the regulation of multiple ionic conductances. In the present study, we recorded at a consistent time of day to control the diurnal input to AVPV kisspeptin neurons. One prominent difference between cycle stages is the sex steroid milieu, especially estradiol, which regulates kisspeptin mRNA and cFos expression (Smith et al., 2006). Specific steroid manipulations suggest a dominant role for estradiol in increased current density of *I*_T_, *I*_h_, and *I*_NaP_, adding strong mechanistic support for estradiol regulation of AVPV kisspeptin neuron firing (Kumar et al., 2015). Estradiol may act through genomic mechanisms to change ion channel expression or function. For example, high estradiol treatment increased the relative levels of Ca_v_3.1 mRNA expression compared with low-estradiol treatment in pools of isolated AVPV kisspeptin neurons (Zhang et al., 2015), although the question remains of whether more cells are expressing Ca_v_3.1 mRNA or the cells increased Ca_v_3.1 mRNA overall. Additionally, estradiol may function via membrane-associated mechanisms to induce post-translational modifications of ion channels or insertion/removal into/from the membrane (Morissette et al., 2008; Vasudevan and Pfaff, 2008). Recently, a genetic tracing approach suggested that only AVPV kisspeptin neurons express the ERα synapse on GnRH neurons (Kumar et al., 2015), further supporting the postulate of an estradiol–kisspeptin–GnRH feedback loop.

In summary, our findings demonstrate that increased firing rate and burst events in AVPV kisspeptin neurons on proestrus versus diestrus likely result from cycle-dependent changes in estradiol modifying multiple conductances. Burst generation may be attributed to the interplay of intrinsic properties with both excitatory input and release from inhibition. Our results support previous observations and extend these by focusing on the regulation of the interactions among intrinsic properties in generating the electrophysiological output of AVPV kisspeptin neurons. The changes in this output associated with the shift between estradiol-negative and estradiol-positive feedback actions likely play an important role in female fertility.

## Synthesis

The decision was a result of the Reviewing Editor Quentin Pittman and the peer reviewers coming together and discussing their recommendations until a consensus was reached. A fact-based synthesis statement explaining their decision and outlining what is needed to prepare a revision is listed below. The following reviewer(s) agreed to reveal their identity: Martin Kelly

A reviewer who previously reviewed your manuscript , as well as a new reviewer have had a chance to look at your manuscript and I am pleased to tell you that their comments are favorable. They agree that you have addressed the major concerns of the previous reviewers for The Journal of Neuroscience. There are a few items that require your attention as follows:

1) For the experiment where the number of rebound spikes is analyzed before and after ZD7288, the actual data should be reported and statistical analysis should be performed.

2) The estradiol link with 4-AP sensitive currents very unclear. All of the data up to this point investigate estrus cycle dependent changes in ionic currents, however, the effect of estrus cycle on 4-AP sensitive currents is not tested. Therefore, the reviewers question if there is support for showing in Figure 9 that IA increases in diestrus and decreases in proestrus.

3) In the discussion, the role of Ih seems to be downplayed. It is clear that modulation of Ih alone does not have a large impact on burst firing. However, it is highly likely that Ih along with the other currents tested here work together to regulated both the magnitude and timing of rebound bursting. This should be emphasized more in the discussion.

Minor editorial matters:

1. In the Discussion, line 660, there is an article missing. It should read "although the question remains....

2. In Figure 9, "Ih" should be labeled properly for the proestrus, high estradiol state.
